# Linoleic and oleic acids enhance cell migration by altering the dynamics of microtubules and the remodeling of the actin cytoskeleton at the leading edge

**DOI:** 10.1038/s41598-021-94399-8

**Published:** 2021-07-22

**Authors:** M. Masner, N. Lujea, M. Bisbal, C. Acosta, Patricia Kunda

**Affiliations:** 1 Centro de Investigación en Medicina Traslacional “Severo Amuchástegui” (CIMETSA), Instituto Universitario Ciencias Biomédicas Córdoba (IUCBC), Naciones Unidas 420, Córdoba, Argentina; 2 Instituto de Investigación Médica Mercedes y Martín Ferreyra, INIMEC-CONICET-Universidad Nacional de Córdoba, Córdoba, Argentina; 3 Instituto de Histología y Embriología de Mendoza (IHEM), Facultad de Ciencias Médicas, Universidad Nacional de Cuyo, Mendoza, Argentina

**Keywords:** Cancer, Cell biology

## Abstract

Fatty acids (FA) have a multitude of biological actions on living cells. A target of their action is cell motility, a process of critical importance during cancer cell dissemination. Here, we studied the effect of unsaturated FA on ovarian cancer cell migration in vitro and its role in regulating cytoskeleton structures that are essential for cell motility. Scratch wound assays on human ovary cancer SKOV-3 cell monolayers revealed that low doses (16 μM) of linoleic acid (LA, 18:2 ω6) and oleic acid (OA; 18:1 ω9) promoted migration, while α-linolenic acid (ALA, 18:3 ω3), showed a migration rate similar to that of the control group. Single cell tracking demonstrated that LA and OA-treated cells migrated faster and were more orientated towards the wound closure than control. In vitro addition of those FA resulted in an increased number, length and protrusion speed of filopodia and also in a prominent and dynamic lamellipodia at the cell leading edge. Using time-lapse video-microscopy and FRAP we observed an increase in both the speed and frequency of actin waves associated with more mobile actin and augmented Rac1 activity. We also observed that FA induced microtubule-organizing center (MTOC)-orientation towards the cell front and affected the dynamics of microtubules (MT) in the direction of cell migration. We propose that environmental cues such as OA and LA present in ascitic fluid, should be taken into account as key factors for the regulation of cell migration.

## Introduction

Cell migration occurs normally during embryonic development, the immune response and wound healing, but also under pathological conditions, like cancer dissemination. Therefore, understanding the nature of factors and mechanisms directing migration is important to fully uncover its role in normal and pathological conditions^1,2^.

During migration, cells establish a front–rear polarity. The remodelling of the cytoskeleton is crucial to establish the structural and functional distinctiveness of both domains. At the cell front, microtubules (MT) and the molecular motors are essential to deliver signaling molecules, mRNAs and membrane cargo to the cell front of the migrating cell^3^. Simultaneously, the structure and dynamic of the actin cytoskeleton (both crucial for cell orientation) combine together to generate the pushing forces that allows the cell to advance^4–6^. At the rear end of a migrating cell, the contractility of actomyosin, regulated by MT, is both required and needed for cell translocation. In both domains, the front and the rear of a polarized migrating cell, several regulators modulate and coordinate the MT network and the activity of actin allowing them to drive cell migration^3^.

It is a well-established fact that extracellular signals modify cell migration. We focused on FA because they are components of the plasma membrane and some of their derivatives have been implicated in controlling cellular functions such as proliferation, apoptosis, migration^7^ and carcinogenesis^8,9^. Particularly relevant are ALA, LA and OA as they are an important and integral part of the diet of mammals. ALA and LA are essential polyunsaturated FA that cannot be synthesized from other precursors in the body thus they have to be ingested. OA is a monounsaturated FA that synthesized in the body via the action of the enzyme stearoyl-CoA 9-desaturase acting on stearoyl-CoA^10^. Another FA of potential interest is arachidonic acid (AA, 20:4 ω6). AA is manufactured in vast amounts by almost all cells in the body and has an impressive array of functions. These two factors make AA a highly ubiquitous molecule that has been extensively studied. Alteration in the levels of AA leads to numerous changes at the cell membrane that makes it very hard to assess its effects. Also, AA and its many naturally occurring analogues show a surprising lack of specificity of action^11^. For these reasons, we did not examine it in the present study.

FA incorporation into the plasma membrane has been shown to affect membrane fluidity, receptor activation and protein function^12–14^. Although previous work reported that some ω3 FA (such as eicosapentaenoic acid and docosahexaenoic acid) may influence cell adhesion, invasion and migration through regulating the polymerization of the actin cytoskeleton (e.g.^15,16^), the role of ALA, LA and OA in cytoskeleton remodeling during cell migration have been less studied.

Here, we hypothesize that addition of ALA, LA and OA could alter the dynamics of actin and MT thereby affecting cell migration. To test this, we used the epithelial ovarian cancer cell line SKOV-3 as a model and wound healing on cell monolayers. We carried out FRET to examine the activity of RhoGTPases and high-resolution confocal video-microscopy to quantitatively explore the effects that addition of the aforementioned FA had on cytoskeleton remodeling during cell migration.

## Results

### LA and OA increase cell migration in SKOV-3 cells

A viability assay using the resazurin method was performed on a cell monolayer with different concentration of ALA, LA and OA (4, 8, 16, 32, 64, 125 and 250 μM)^17^ to determine the non-cytotoxic dose for each FA. Ethanol was used as control (Suppl. S1a). Viability of each experimental condition was expressed as relative to control (100%). Cell viability dropped significantly at FA concentration higher than 32 μM for LA and ALA and higher than 125 μM for OA. We chose 16 μM for all three FA in the next series of experiments, as this was the highest concentration that showed no cytotoxicity for any of the 3 FA tested and slightly higher concentrations were reported as cytostatic in previous publications^18,19^.

Cell proliferation is of concern in migration experiments. To test its relevance in our setting, we analyzed mitosis on a cell monolayer treated with 16 μM of each FA for 24 h. We used an anti-phospho Histone-3 (Ser-10) antibody as a mitotic marker and DAPI to label nuclei on fixed monolayers^20^. The results showed that 16 μM had no effect on cell proliferation relative to control (Suppl. S1b-c).

**Figure 1:**
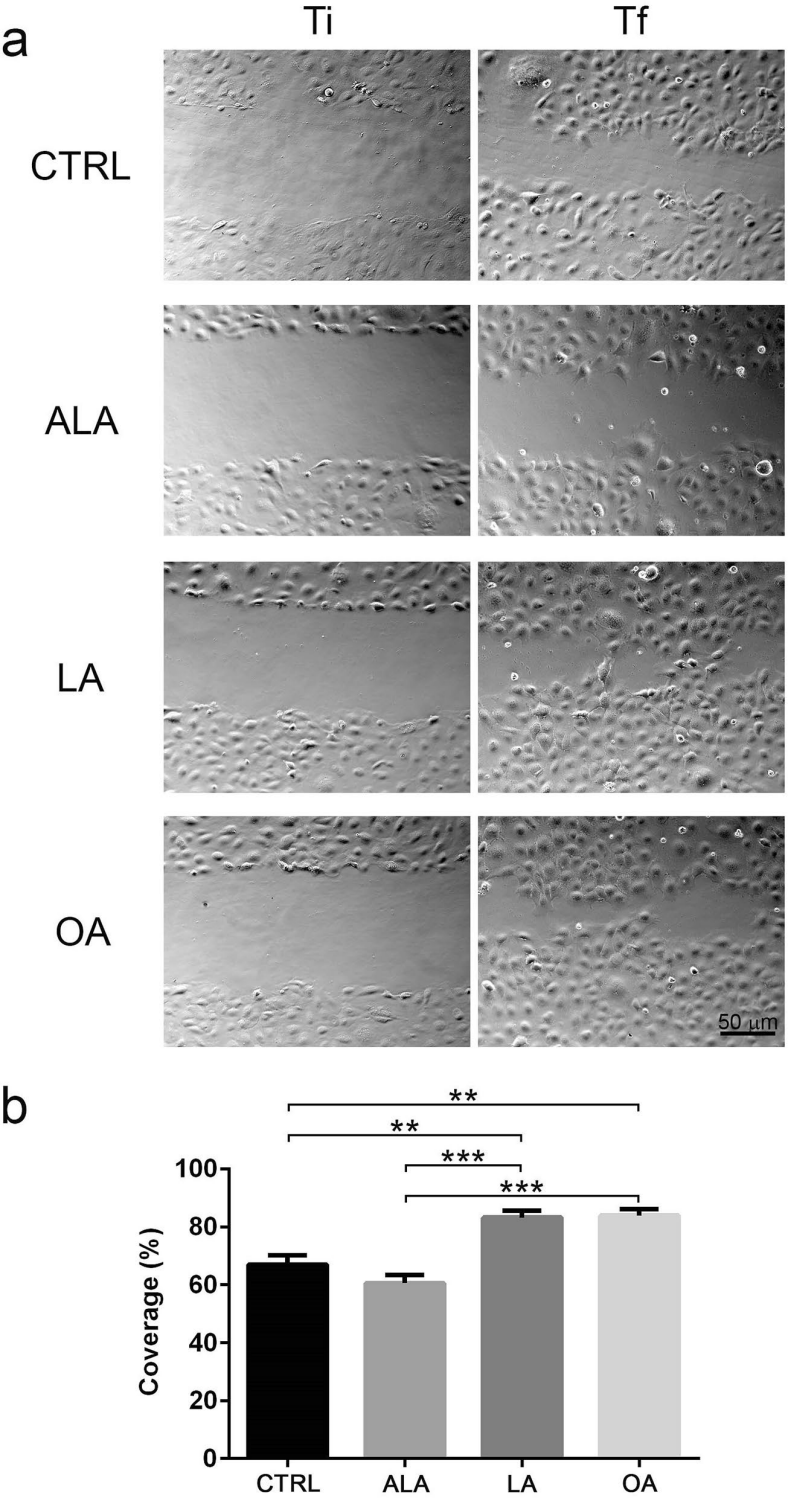
LA and OA promoted SKOV-3 cell migration in an in vitro wound-healing assay. (**a**) Phase microscopy of the scratch zone within the first 2 h after scratch (Ti) or at 24 h after the scratch (Tf) for treatment with serum free media containing ethanol (control), ALA, LA or OA. (**b**) Relative coverage of the wound area expressed as percentages. Graph shows mean ± SEM of 10 photographs per condition in 4 independent experiments. Asterisks represent statistical significance using Kruskal–Wallis test with Dunn’s multiple comparisons (**p < 0.01, ***p < 0.001). Scale bar 50 μm.

To study the effect of ALA, LA and OA on cell migration, we performed a scratch-wound assay on a cell monolayer. Phase images were taken at 2 (initial time or Ti) and at 24 h (final time or Tf) after the scratch (Fig. 1a). The results showed that LA and OA increased migration by ~ 20% on average compared to control untreated cells (N = 4, p < 0.01). ALA treated cells migrated slightly less than control, although it was not statistically different (Fig. 1b).

These results together suggested that addition of LA or OA directly affects cell migration in a scratch wound assay. Because ALA data did not differ significantly from control, we focused our further analysis on LA and OA.

### LA and OA affect the orientation polarity and cell trajectory during migration

**Figure 2:**
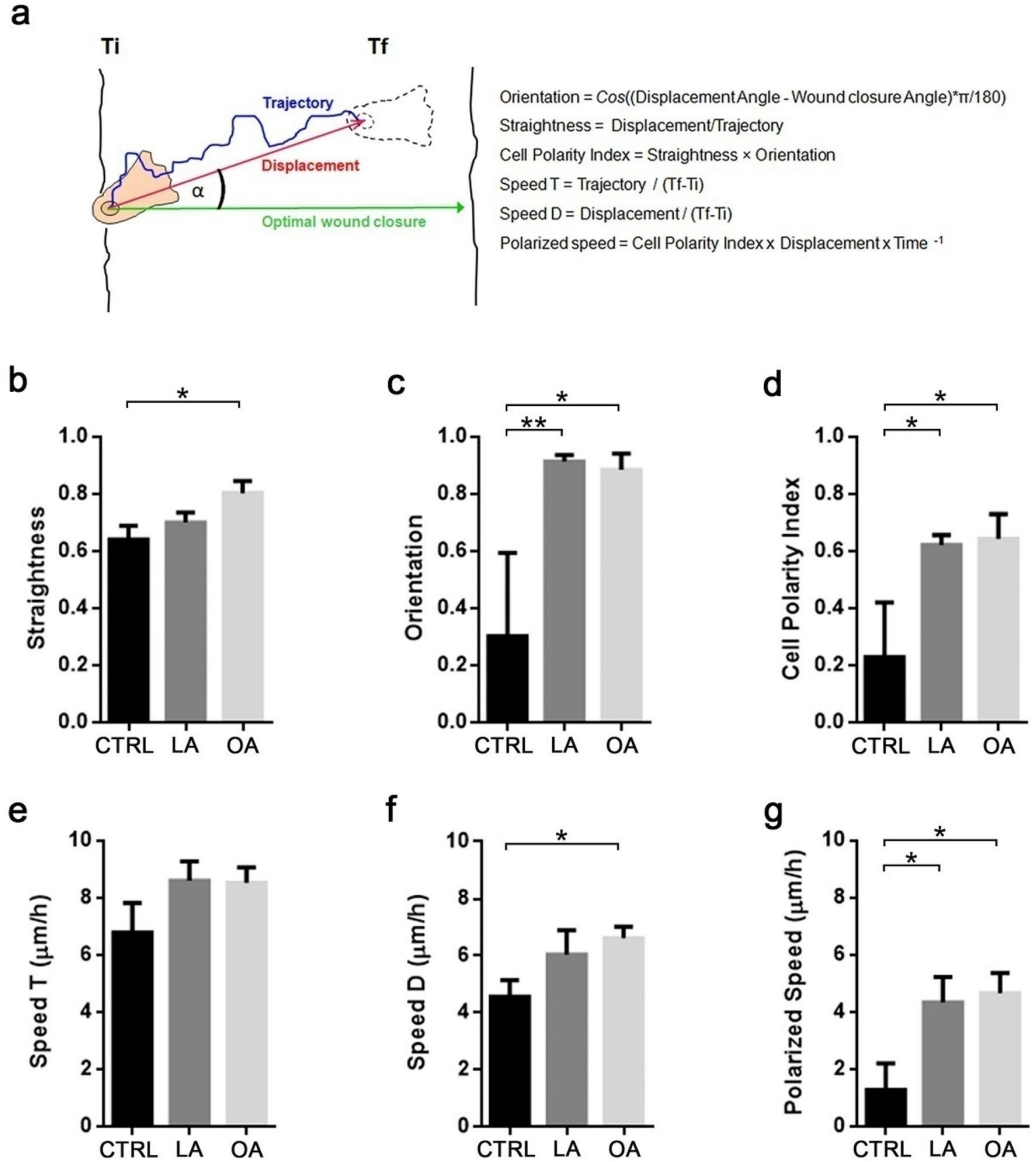
LA and OA enhanced cell orientation and polarity during migration. (**a**) The diagram depicts the variables measured and calculated of cell movements during the time lapse of the video imaging: straightness (**b**), orientation (**c**) and cell polarity index (**d**). Speed taking into account the cell trajectory (**e**), displacement (**f**) and integrative index polarized speed (**g**). Plots show mean ± SEM and asterisks represent statistically significant differences by Students t-test (*p < 0.05, **p < 0.01). Data obtained from 10 to 15 trackings per condition.

We performed a phase contrast time-lapse videomicroscopy to visualize the effect of LA and OA on the migration in a scratch wound assay (Fig. 2). The monolayer was imaged for up to 8 h after scratch. We took into consideration cell trajectory, displacement and the angle of migration in relation to the optimal wound closure direction (see “Methods”) to quantitatively analyze the migratory pattern of treated and untreated cells. An explanatory diagram of the variables is shown in Fig. 2a.

Image analyses in our setting showed that cells migrated using a straight trajectory towards the wound, evinced as a significant increment in straightness in OA (control 0.64 ± 0.05, n = 6; OA 0.80 ± 0.04, n = 11; p < 0.05) with no differences in LA relative to control (control 0.64 ± 0.05, n = 6; OA 0.70 ± 0.03, n = 12) (Fig. 2b). The comparative orientation in both LA (0.91 ± 0.02, n = 12; p < 0.01) and OA (0.88 ± 0.05, n = 10; p < 0.05) were significantly higher by threefold relative to control (0.30 ± 0.29, n = 6) (Fig. 2c). Accordingly, we further evaluated a cell polarity index. Data showed that the cell polarity increased significantly for LA and OA treated cells (LA 0.62 ± 0.03; OA 0.64 ± 0.09) compared with control (0.23 ± 0.19) (p < 0.05) (Fig. 2d).

We also analyzed the speed T that considers the trajectory that the cell followed per h (Fig. 2e) and speed D that consider cell displacement (Fig. 2f). Speed T in LA (8.60 ± 0.68 μm/h) and OA (8.52 ± 0.54 μm/h) showed no difference in relation to control (6.78 ± 1.03 μm/h). On the contrary, speed D increased significantly for OA (6.61 ± 0.40 μm/h; p < 0.05) compared with control (4.56 ± 0.57 μm/h), with a non-significant increment for LA (6.04 ± 0.85 μm/h, ns) (Fig. 2f). We also calculated a polarized speed (Fig. 2g) that represents the effective speed at which the cell moves in the optimal direction towards wound closure. The data showed that polarized speed in LA and OA exhibited a significant fourfold increment compared with control untreated cells (control 1.28 ± 0.92 μm/h; LA 4.34 ± 0.88 μm/h; OA 4.66 ± 0.69 μm/h; p < 0.05). Taking together, these results show that LA and OA treated cells migrate at similar speed than control, however, they follow a straight and polarized trajectory optimally orientated to the closure of the wound.

### LA and OA do not affect morphometric patterns in treated cells

To explore how FA increased the migration in a cell monolayer we analyzed actin structures in SKOV-3 cells treated with LA and OA for 24 h. During migration cells can form at least four different plasma membrane protrusions at the leading edge: lamellipodia, filopodia, blebs and invadopodia, with different contributions to migration.

Actin staining with phalloidin-TRITC revealed that cells exhibited polarization at the edge towards the front of migration 2 h after scratching (Suppl. S2). Representative SKOV-3 cells stably expressing LifeAct-GFP also showed a polarized lamellipodia at the cell front at 2–4 h after scratching (Suppl. S2b).

Because multiple cell fronts might reduce cell polarity and movement in contrast to the presence of a unique main protrusion that may facilitate migration efficiency^21,22^, we measured the number of leading edges and their perimeter at the cell front. Our data showed no differences between control and FA treated cells (Suppl. S2a) regarding either the number of leading edges (~ 1 per cell, Suppl. S2b,c), or leading edge perimeter (40–60 μm per cell front Suppl. S2d).

Given that cell cortical tension affects the ability of a cell to migrate, we performed a quantitative analysis of cell spreading as a proxy for cortical tension^20,23^. The data showed no significant differences between control and FA treated cells regarding cell perimeter, area, circularity and roundness (Suppl. S3a–d). In addition, we found no statistically significant differences in cell adhesion relative to control in LA or OA treated cells (Suppl. S3e).

### OA and LA-treatments increase various parameters of filopodial dynamics at the leading edge during migration

**Figure 3:**
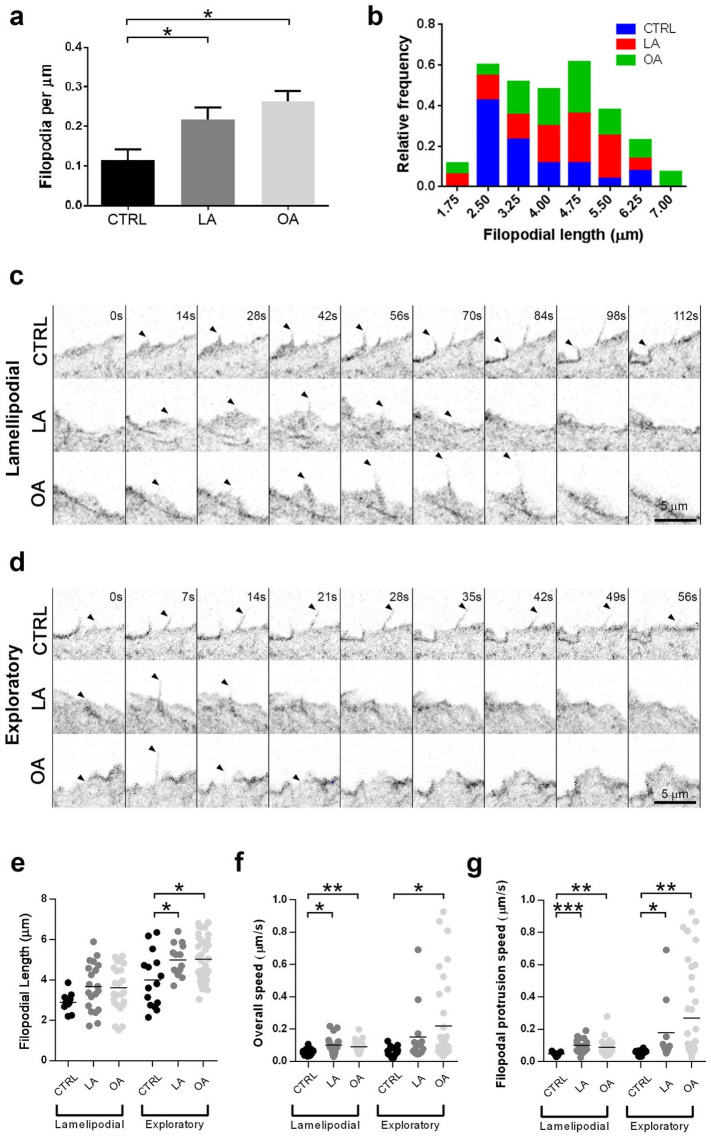
OA and LA altered actin-based structures at the leading during migration. (**a**) Mean number of filopodia per μm at the leading edge in Control (black), LA (gray) and OA (light gray) treated cells, significance tested with Kruskal–Wallis test and Dunn's multiple comparisons, n = 15–45 per subgroup in 2 independent experiments. (**b**) A histogram shows the overall change in distribution of filopodial length in control (blue), LA (red) and OA (green) cells. (**c**,**d**) Still images from a time-lapse videomicroscopy depicting lamellipodicfilopodia (**c**) and exploratory filopodia (**d**) in SKOV-3 LifeAct-GFP cells for the control group and cells treated with LA or OA. The time of each frame is shown on the top of the control panel. The arrowhead points out a filopodia that completes its protrusion cycle within the shown timeframe. Scale bar 5 μm. (**e**) Filopodial length comparison among treatments. (**f**) Filopodial speed reflects the time (expressed in μm/s) that the filopodia takes to protrude and retract. (**g**) Filopodia protrusion speed reflects the time (expressed in μm/s) that the filopodia took to protrude. Data presented as scatter dot plots with a line at the mean value, significance tested with Kruskal–Wallis test and Dunn's multiple comparisons, n = 15–45 per subgroup, 2 independent experiments. Asterisks represent statistical significance (*p < 0.05, **p < 0.01, ***p < 0.001).

Filopodia are one of the actin-based structures with an important role in cell migration^21,22^. Quantitative analysis of still images showed that LA and OA treated cells have two and three times more filopodia than control cells at the leading edge, respectively (0.10 in control vs. ~ 0.25 in LA and 0.28 in OA, p < 0.05) (Fig. 3a). While control cells had a higher proportion of short filopodia (mode = 2.5 μm), both the higher proportion in LA (mode = 4.75 μm) and OA (mode = 4.75 μm) showed longer filopodia than control (Fig. 3b). In addition, OA showed some filopodia that reach 7 μm of length (Fig. 3b, Suppl. S4b) representing the longest among the longer filopodia in control and LA.

We performed time-lapse confocal videomicroscopy on SKOV-3 cells stably expressing LifeAct-GFP to analyze filopodial dynamics (Movies 1–3; Fig. 3c,d). For quantization we only considered filopodia that grew and retracted during image acquisition. We distinguished 2 types of filopodia: those that protruded and then retracted from the leading edge (named “exploratory filopodia”) (Fig. 3c); and those in which the filopodium emerged embedded in a lamellipodium, therefore named “lamellipodic filopodia” (Fig. 3d).

On average, in absence of FA, we found that lamellipodic filopodia were significantly shorter than exploratory filopodia (Lam 2.89 ± 0.14 μm, n = 11; Exp 4.00 ± 0.35 μm, n = 15; p < 0.05) (Fig. 3e). However, lamellipodic filopodia length between control and FA treated cells did not significant differed (control 2.89 ± 0.14 μm, n = 11; LA 3.67 ± 0.26 μm, n = 20; OA 3.61 ± 0.22 μm, n = 25).

In addition, we found a similar length for exploratory filopodia in LA (4.99 ± 0.21 μm, n = 14) and OA (5.03 ± 0.19 μm, n = 37; p < 0.05) with both longer than control (4.00 ± 0.35 μm, n = 15).

In control cells lamellipodic filopodia length ranged from 2.2 to 3.9 μm, LA from 1.7 to 5.9 μm and OA from 1.5 to 5.2 μm. In addition, exploratory filopodia were longer in LA (albeit not significantly) and OA (p < 0.05) than control. Moreover, control cells had a higher range of length (from 2.1 to 6.4 μm) than treated cells (LA 3.7 to 6.4 μm and OA 3.4 to 6.9 μm). Furthermore, FA treatment reduced the overall amount of short exploratory filopodia (Fig. 3b–e). We concluded that FA had an effect on exploratory filopodia, shifting the size distribution of the shortest filopodia present in control to longer filopodia in both LA and OA (Fig. 3b).

For dynamic analysis we quantified two speeds: the overall speed that considers both the filopodia protrusion and retraction time; and the filopodial protrusion speed that takes into account only the filopodial protrusion time. The latter discount the time the filopodia remains elongated. The quantization of the overall speed showed that both lamellipodic (control 0.05 ± 0.01 μm/s, n = 13; LA 0.10 ± 0,01 μm/s, n = 20; p < 0.05; OA 0.09 ± 0.01 μm/s, n = 25; p < 0.01) and exploratory filopodia (control 0.07 ± 0.01 μm/s, n = 14; LA 0.15 ± 0.05 μm/s, n = 14; OA 0.22 ± 0.04 μm/s, n = 37; p < 0.05) were faster in LA and OA than control untreated cells (Fig. 3f). Moreover, the analysis revealed that LA and OA induced an increment in the filopodia protrusion speed compared with control in both lamellipodic (control 0.05 ± 0.01 μm/s, n = 13; LA 0.10 ± 0.01 μm/s, n = 20; p < 0.001; OA 0.09 ± 0.01 μm/s, n = 25; p < 0.01) and exploratory filopodia (control 0.056 ± 0.005 μm/s, n = 12; LA 0.180 ± 0.060 μm/s, n = 10; p < 0.05; OA 0.27 ± 0.05 μm/s, n = 37; p < 0.01) as shown in Fig. 3g. In addition, LA and OA contained some exploratory filopodia with a speed notably faster than the fastest filopodia in control reaching velocities near 1 μm/s (Suppl. S4).

To characterize the fastest filopodia subpopulation we examined the correlation between filopodial speed and length (Suppl. S4). We found a positive correlation between speed and length in lamellipodic filopodia in both LA (r_s_ =  + 0.75; p < 0.001) and OA (r_s_ =  + 0.74; p < 0.01) (Suppl. S4a) but not in control. Albeit we found no significant correlation between length and speed for the whole set of exploratory filopodia, we identified a longer and faster exploratory filopodia subpopulation that exhibited a significantly positive correlation between length and speed for OA (and to a lesser extent for LA) (r_s_ =  + 0.72; p < 0.05) (Suppl. S4b).

Taking all these results together, dynamic analysis showed that LA and OA treated cells form more filopodia at the leading edge during migration. It also showed the presence of longer exploratory filopodiain OA treated cells. Additionally, we identified a subpopulation of filopodia that were longer and faster than control that exhibited a “lashing” behavior in the presence of OA.

### LA and OA induced an increment of actin dynamic at the migration cell front

**Figure 4:**
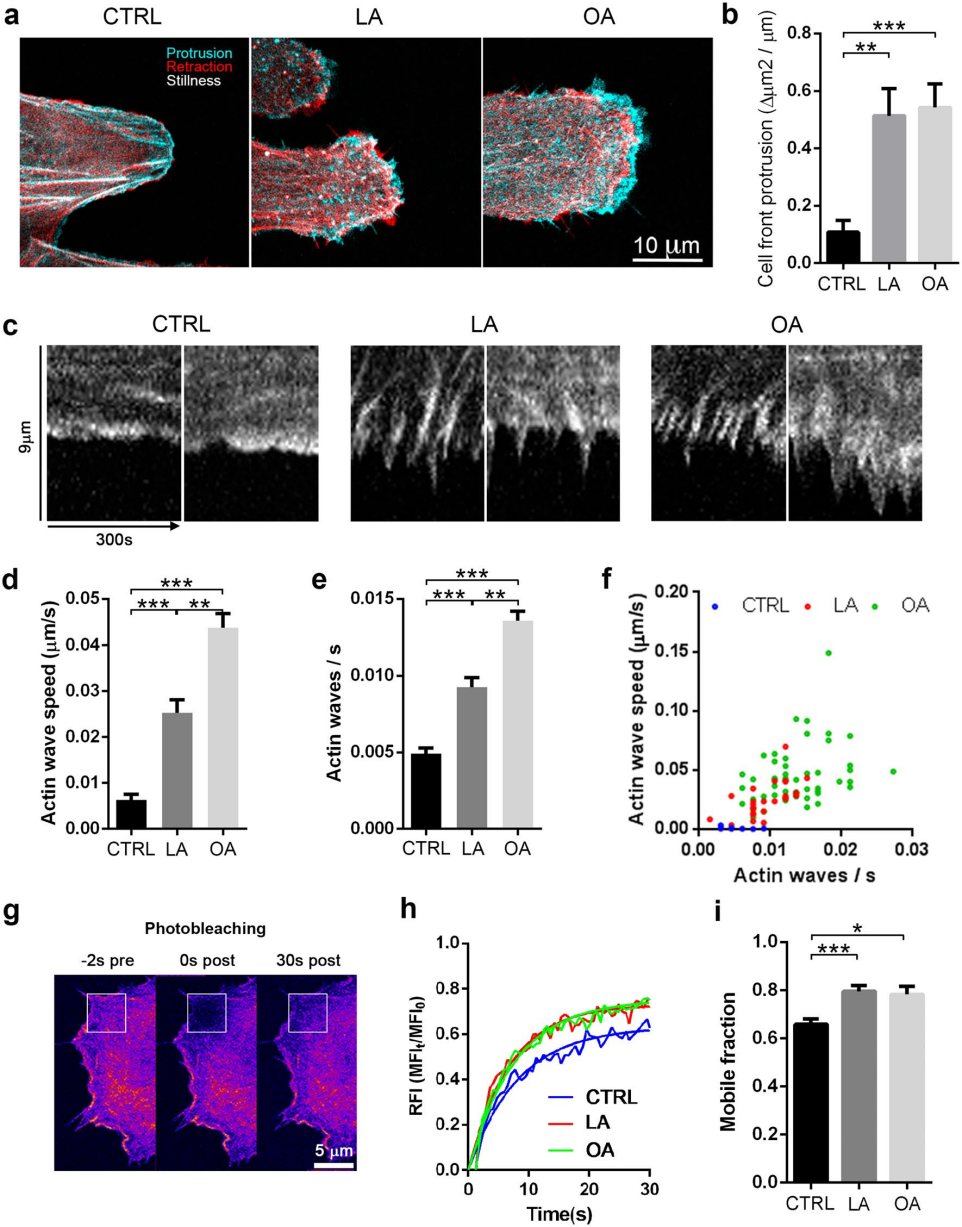
LA and OA increased the structure dynamic of actin at the migration cell front. (**a**) Superposition of confocal images of leading edges of live SKOV-3 LifeAct-GFP cells treated with vehicle, LA or OA, 4–6 h post scratch. Cyan represents the advance at the front, red shows retraction, and still actin structures are shown in white. Scale bar 10 μm. (**b**) The plots show the accumulated protrusions as obtained by superposition of images over a 5 min interval relative to the leading edge perimeter for each cell. (**c**) Kymographs of the leading edge during 5 min of time-lapse (two representative images per condition). Plots showing the mean ± SEM of (**d**) Actin wave retrograde speed and (**e**) wave frequency. (**f**) Correlation analysis shows that wave frequency and retrograde speed are positively correlated for LA and OA. (**g**) FRAP on the leading edge of the SKOV-3 LifeAct-GFP cells at three times: actin pre-photobleaching on the cell border (left), immediately after photobleaching (middle) and 30 s after the photobleaching, thus revealing recovery of fluorescence-signal (right). Scale bar 5 μm. (**h**) Exponential recovery curves from control (blue), LA (red) and OA (green) treated cells, and recovery rate was analytically obtained from the one-exponential function that best fitted the data. (**i**) Graph showing the mean ± SEM of the mobile fraction obtained from the FRAP experiments. Asterisks represent statistical significance (*p < 0.05, **p < 0.01, ***p < 0.001).

We further analyzed the lamellipodia at the leading edge (Fig. 4). Images superposition from a time lapse of SKOV-3 LifeAct-GFP showed the net lamellipodia advance in red, lamellipodia retraction in blue and the lack of movement (stillness) in white, during the time acquisition (Fig. 4a). The dynamic analysis of the lamellipodia area showed that LA (0.51 ± 0.09 μm, n = 11; p < 0.01) and OA (0.54 ± 0.08 μm, n = 17; p < 0.001) treated cells advanced 5 times more than control at 8 h post scratching (0.11 ± 0.04 μm, n = 11) (movies 1–3; Fig. 4a,b).

Directional assembly–disassembly of actin generates a movement of wave-like membrane protrusions measured as retrograde flow. The speed of the retrograde flow correlates with the establishment of cellular polarity and subsequent migration^24^. Therefore, we wanted to quantitatively ascertain whether or not LA and OA addition had any influence on the retrograde flow of actin.

We generated a kymograph using time lapse confocal video microscopy on the lamellipodia at the cell front in each experimental condition (Fig. 4c). The kymograph showed that the rearward actin flow behaves in fact as waves (as described in Yamashiro and Watanabe 2014)^24^. Our analysis revealed that LA and OA increased the actin waves speed compared with control (control 0.0060 ± 0.0001 μm/s, n = 29; LA 0.025 ± 0.003 μm/s, n = 26; p < 0.001; OA 0.044 ± 0.003 μm/s, n = 57; p < 0.001), with some differences between LA and OA (p < 0.01) (Fig. 4c,d). Also OA and LA increased the actin waves frequency (in waves per second) compared to control (control 0.0050 ± 0.0004 waves/s; LA 0.0090 ± 0.0006 waves/s; p < 0.001; OA 0.014 ± 0.0006 waves/s; p < 0.001) (Fig. 4e). Correlation analysis between wave speed and frequency revealed that a higher speed translated into a higher frequency in LA and OA treated cell during cell migration (for control r_s_ =  + 0.04; LA r_s_ =  + 0.70; p < 0.001; OAr_s_ =  + 0.33; p < 0.05) (Fig. 4f), albeit the control group failed to exhibit any relationship.

We explored further whether or not FA addition modified actin turnover using fluorescence recovery after photobleaching (FRAP). We used a high intensity laser on the front of migration of SKOV-3 LifeAct-GFP cells, and the fluorescence recovery of the bleached area was observed over 30 s at a 0.4 s interval. Figure 4g shows a representative cell before and after photobleaching and post-photobleaching recovery. The fluorescence intensity profile over time fitted an exponential recovery curve (Fig. 4h). The mobile fraction of actin in control group plateaued near 60%, whereas LA and OA treated cells reached significantly higher values (LA 75%, p < 0.001, OA 90%; p < 0.05) (Fig. 4i). In short, LA and OA increased the mobile actin fraction, thus affecting the dynamics of actin.

### OA induced a differential activation of RhoGTPases at the front and rear of the migrating cell

**Figure 5:**
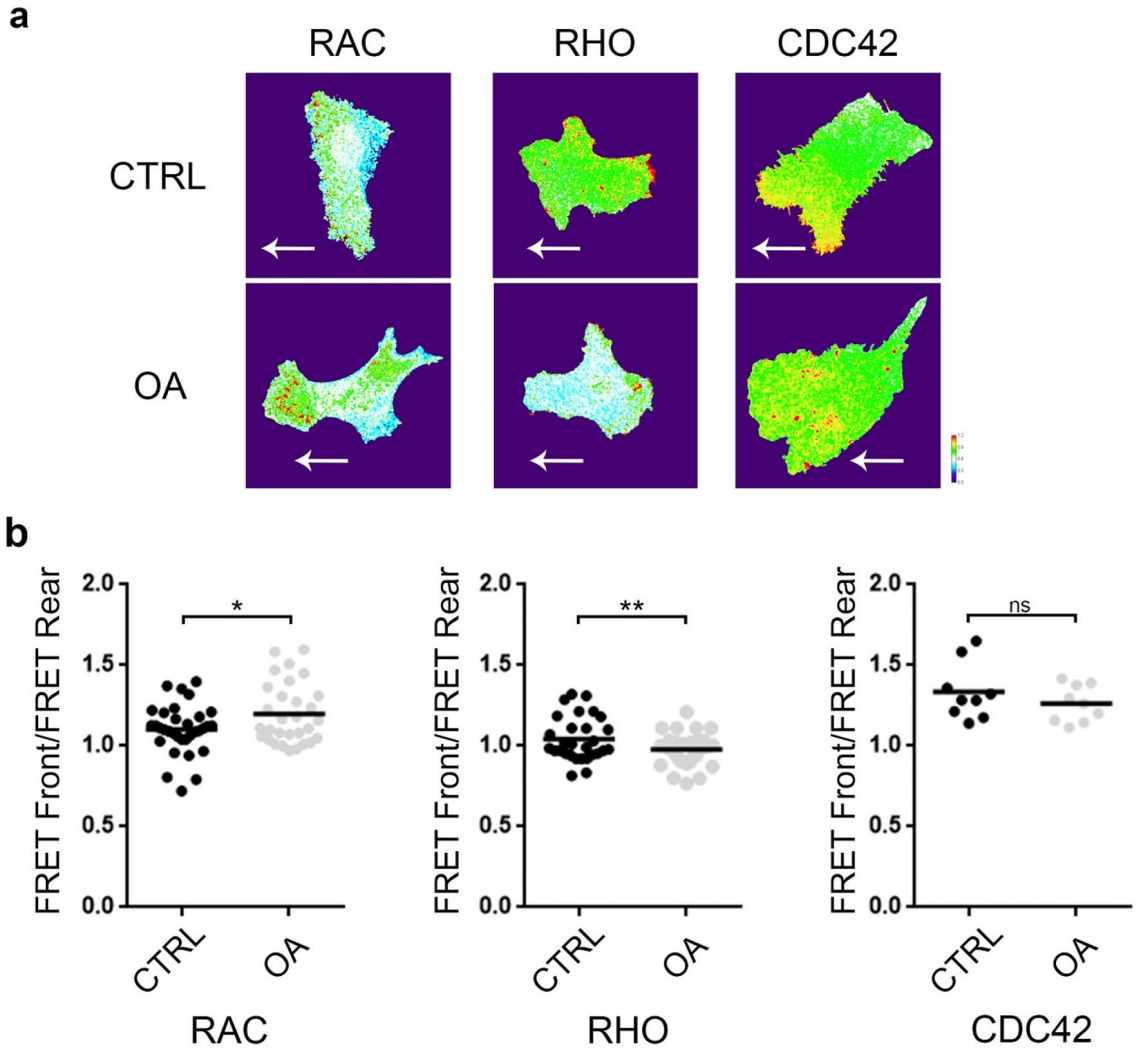
FRET revealed that OA affect the activity of RhoGTPases in migrating cells. (**a**) Confocal images of representative cells showing FRET signals for Rho, Rac and Cdc42 FRET probes in control (top) and OA (bottom). The white arrow indicates the orientation of the cell during migration. Scale bar 20 μm. (**b**) Scatter plot of the Front/Rear ratio of FRET from three independent experiments. The statistical significance was tested using an unpaired t-test. *p < 0.05, **p < 0.01.

To test the effect of OA on RhoGTPases activity we set up a FRET analysis using FRET probes for Rho, Rac and Cdc42 (Fig. 5). We performed FRET calculations (see “Methods”) at the front and the rear of the migrating cell and reported here the ratio between the two. Only the first line of cells, that is the closest to the scratch, was examined. We chose the ratio front/rear (F/R) in each cell to limit the impact of inter-cell variations generated by differences in the expression levels of the probes or bleaching during the confocal acquisition. The Fig. 5a shows representative cells for each probe in OA and control. The average F/R ratio of FRET for Rac significantly increased with OA treatment (control 1.10 ± 0.02, n = 33; OA 1.20 ± 0.03, n = 30; p < 0.05). The F/R ratio of FRET for Rho decreased in OA compared with control (1.03 ± 0.02, n = 43 vs. OA 0.96 ± 0.02, n = 50; p < 0.005) (Fig. 5b). Albeit FRET for Cdc42 decreased in OA treatment both at the cell front and the rear, the F/R ratio did not change significantly (control 1.33 ± 0.06, n = 9; OA 1.26 ± 0.04, n = 9) (Fig. 5b).

The results suggest that OA influences RhoGTPase activity modifying differentially the activity of Rac, Rho and Cdc42 in a migrating cell.

### Addition of OA oriented Microtubules towards the front of migration

For cells to migrate with a preferred orientation and direction, we would expect that FA also alter microtubules orientation.

**Figure 6:**
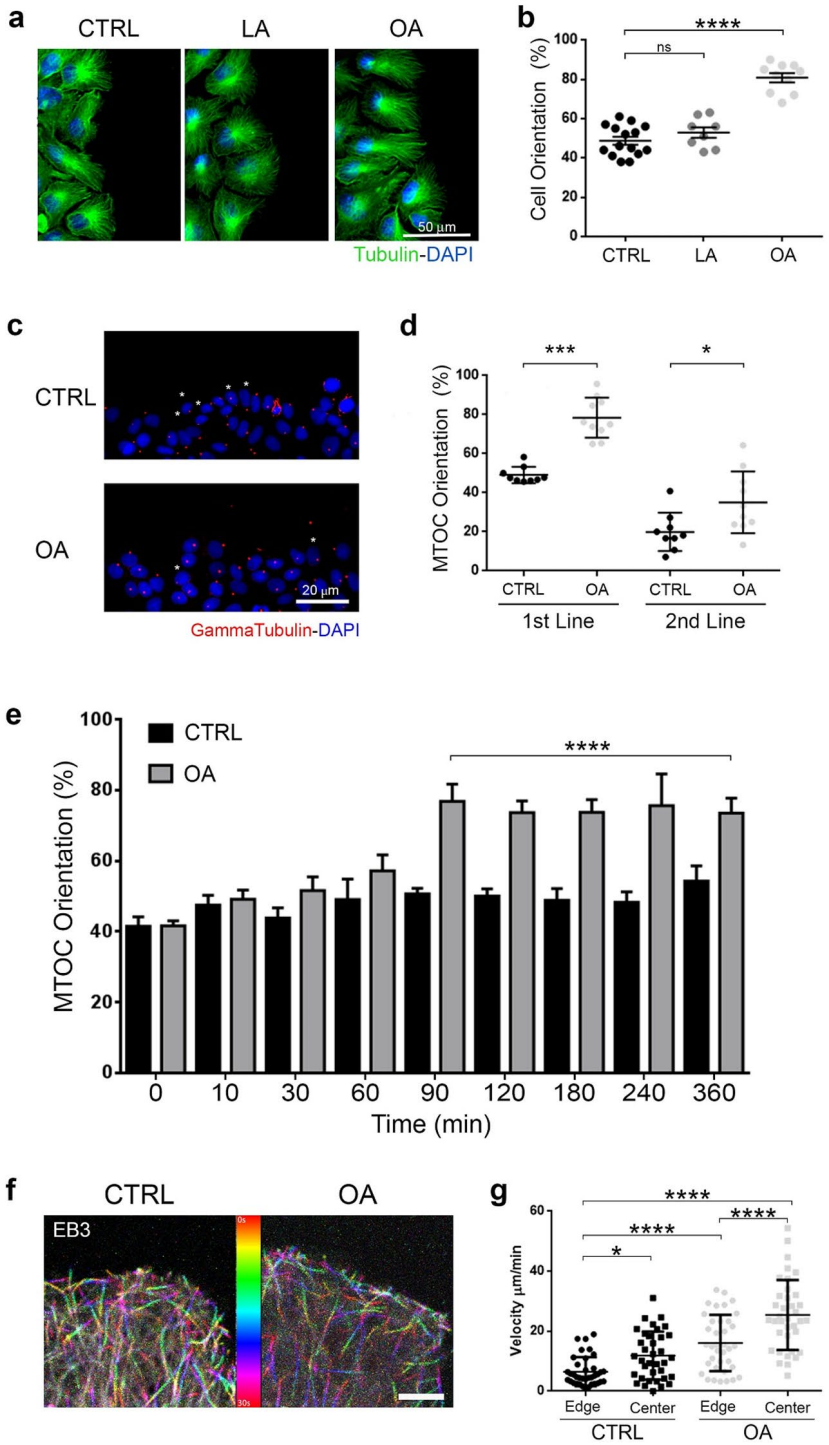
OA orientated microtubules toward the migration front. (**a**) Tubulin (green) and DAPI (blue) labeling of SKOV-3 cell monolayers treated for 24 h with LA, OA or ethanol (vehicle), 2 h after scratching. Scale bar 50 μm. (**b**) The plot shows the proportion of polarized cells in the presence of LA, OA and control as evaluated by orientation of alpha-tubulin. (**c**) Gamma tubulin (red) and DAPI (blue) labeling of SKOV-3 cell monolayer treated for 24 h with OA (bottom) or ethanol (top), at 0 h (top) and 2 h (bottom) after scratching. Asterisks show non polarized cells. Scale bar 20 μm. (**d**) Scatter plot of the proportion of MTOC-orientation in the direction of the closure of the wound for the first and the second line of cell in the monolayer. Data from 3 independent experiments. Asterisks represent statistical significance (*p < 0.05, ***p < 0.001). (**e**) The graph shows the mean ± SEM (n = 3) of the MTOC-orientation as evaluated by gamma-tubulin staining at different times after the scratch. (**f**) Representative images of the front of migration of SKOV-3 EB3-Cherry. The image corresponds to a superposition of 60 frames from the time lapse confocal video microscopy in Fiji (https://imagej.net/Fiji RRID:SCR_002285). Scale bar 50 μm. The color bar shows the color code from 0 to 30 s. (**g**) Scatter dot plots of the MT tip velocity from the edge and center of control and OA treated cells. Asterisks represent statistical significance for the Mann–Whitney test; *p < 0.05 and***p < 0.0001.

Therefore we analyzed MT orientation as it plays a key role in polarization and orientation during cell migration (Fig. 6). Alpha-Tubulin immunolabeling revealed that MT are already orientated towards the closure of the wound 2 h after scratching (Fig. 6a). On average, we found about half of the cells in the control group already polarized in the front line (52.88 ± 2.70%, n = 8), while LA did not show differences with control (48.73 ± 2.00%, n = 15) and OA remarkably increased MT orientation (80.80 ± 2.34%, n = 10; p < 0.0001) (Fig. 6b).

To assess the time course of the effect of OA on cell polarity, we evaluated the orientation of the MTOC as this protein complex represents the main contributing factor to establish cell polarity and the formation of a front-orientated microtubule network. We analysed the MTOC orientation at different time points by immunolabeling against gamma-tubulin. MTOC orientation in control was slightly increasing over time although with no significant differences from the time zero up to 6 h post scratching (49.47 ± 3.05%) (Fig. 6c–e). OA induced an increment in MTOC orientation over time and the increment became significant after 90 min post scratching respect to control at the same time (76.84 ± 4.86%, n = 5; p < 0.0001). In addition, once OA reached around 80% MTOC orientation it maintained this value up to 6 h post scratching. Notice that OA had a similar effect on MTOC orientation on the cells situated immediately behind the cells closest to the wound. However, the effect was somewhat smaller albeit statistically significant when compared to control (control 0.20 ± 0.03%, n = 9; OA 0.35 ± 0.05%, n = 10; p < 0.0001) (Fig. 6c,d).

To assert the effect of OA on MT dynamic we quantitatively analyzed MT growth using an EB3-Cherry stable cell line (Fig. 6f). We differentiated comets at the border of the cell and the comets in the cell body and we analyzed those orientated towards the front of cell migration. As it is shown in Fig. 6g, under control conditions MT grew faster in the cell center than in the border of the cell (11.86 ± 1.33 μm/min, n = 36 vs.6.48 ± 0.79 μm/min, n = 37; p < 0.05). This agrees with previous *in vitro* observations^25^. More importantly, OA induced an increment in the speed of MT growth in both locations, in the cell center (OA 25.41 ± 1.91 μm/min, n = 37) and the cell border (OA 16.07 ± 1.535 μm/min, n = 37; p < 0.0001).

Together, our data indicates a marked and early effect of OA on MT orientation and dynamic, not only in the first line of migrating cells in the monolayer but also in the second line of cells. This finding suggests that OA affects the establishment and maintenance of a directed movement potentially on the whole monolayer.

## Discussion

The etiology and progression of several pathological processes such as cancer metastasis, autoimmune disorders, and vascular disease, among others depends on cell migration. Cells respond to a large number of environmental stimuli that facilitate or prevent movement or direct it. Interestingly, metastatic ovarian cancer cells inhabit an environment enriched in factors that are likely to promote cell migration and spread of the disease^26,27^.

To explore this possibility, we chose 3 representative unsaturated fatty acids present in the ascitic fluid of patients with ovarian cancer and analyzed if and how they modify the cellular motility machinery^28^. To achieve this, we added the FA to cultures of SKOV-3 cells and evaluated how their addition altered parameters associated with the cytoskeleton that determines cell motility.

Overall, our results showed that OA and LA induced a migration that is both faster and straighter-orientated. We proposed that this is the result of 2 separate and yet interdependent actions of these FA. Firstly, by triggering a fast MTOC orientation followed by MT orientation towards the cell front coupled with a sustained active dynamic of those MT. Secondly, the other mechanism may involve an increment in the dynamic and remodeling of the actin cytoskeleton in a Rac-dependent manner at the leading edge and a Rho-dependent manner at the rear of the migrating cell. Our data support this model.

In relation to the actin cytoskeleton, we found that cells treated with OA and LA increased not only the number of filopodia but also the proportion of longer filopodia at the leading edge. Although the analysis of the overall filopodial length revealed no correlation with speed in any treatments, LA and OA promoted the emergence of a subpopulation of filopodia that were the longest and the fastest, with both parameters exhibiting a strong correlation. Our work provides the first demonstration that OA induces the formation of long and very fast filopodia. With regards to the lack of correlation between the length and speed of filopodia, we must bear in mind that cytoskeletal protrusions as either lamellipodia or filopodia, need to overcome the backward enveloping force of the plasma membrane (10 pN for a lipid bilayer)^29^ in order to push the limits of the cell during locomotion. Regarding this subject, Bornschlögl concludes that force contributions from membrane tension become an important factor when compared to actin based forces at the cell edge (in which filaments push at the pN order of magnitude)^30^. Then, longer filopodia (even though protruding at the same speed) increase the contact zone with the substrate and generate the higher traction needed for cell movement^30^.

Lamellipodia have been shown to drive cell migration^31–33^. Actin waves in the lamellipodia contribute to the retraction of the actin network, driving the cell body forward. The movement of actin polymers to the back eventually leads to their depolymerization, enabling the recycling of actin that maintains actin polarization at the cell front^34,35^. Mechanical forces arriving from motor proteins like non-muscular myosin help to retract the network^36^.

In our experimental setting, SKOV-3 cells exhibited a lamellipodial protrusion speed and wave activity clearly different between the treatments. The rearward flow of actin from the edge showed wave patterns as described in Yamashiro 2014^24^. LA and OA seemed to induce different dynamics in terms of speed and frequency, which are positively correlated in those groups but not in the control. Increased retrograde flow, as evidenced by the actin waves, could offer an explanation for the increment in the migration capacity in the absence of adhesion differences between treated and non-treated cells. In this regard Yamashiro states that some cells move faster by raising their actin wave frequency, a behavior adopted independently of the maturation state of nascent adhesions to focal adhesions^24^.

In agreement with a higher actin wave activity, our FRAP experiments showed a higher mobile fraction in LA and OA treated cells. Albeit we cannot entirely dismiss an effect of LifeAct-GFP diffusion the temporal resolution of our experiment prevented us from detecting ultra-fast-unbound LifeAct-GFP diffusion. Therefore, we believe that the values observed in FRAP may be an indicator of a higher amount of actin participating in cytoskeletal reorganization at the leading edge, consistent with the increased motility, structural differences and dynamics we observed^37,38^.

The fact that the cells treated with LA and OA migrated following a straighter trajectory made for a more efficient migration that is, covering the same distance in less time. This agrees with Maiuri’s statement that the ability of a cell to persist moving “in a defined direction” universally and directly translates into an effective migration speed in all cell types^39,40^. In that work, increased actin retrograde flow and persistent cell movement resulted in a net speed increment. Our results on trajectory, displacement, polarization, raw speed and polarized speed complement our data on actin dynamics and also agree with current literature.

The ability to maintain the directionality of the displacement we attributed also to both faster orientation of the MTOC plus a more efficient MT orientation in the presence of LA and OA. We observed this effect of OA not only in the first line of cells but also in the line of cells behind that, suggesting two possibilities: one, that OA affects the MT on all the cells of the monolayer or, that the flow of information from the cells at the front to the cells in the rear (also known as “collective migration”) is somewhat improved^21,41^.

In addition, by using the EB3-cherry labelled we observed that OA increased the dynamic of MT at the leading edge, a phenomenon that may well contribute to an enhanced rate of migration.

Our interpretation is that a higher MT dynamic at the migration front could aid the supply of membrane and enhance the recruitment of RhoGTPase activators at the leading edge. In support of this view, we found more activation of Rac at the cell front relative to the rear in OA treated cells. On the other hand, activated Rho decreased in the front compared to the rear of the migrating cell stimulated with OA. Thus, these ups and downs of Rac and Rho could regulate the dynamic of actin at the leading edge. This also suggests the possibility of a direct regulation of Rac and/or Rho by OA. Whether or not this is the case remains to be established in our model. The contribution of MT to the regulation of the assembly and dynamic of actin has been described in other cell models. Furthermore, there are a growing number of regulators of MT dynamic that also regulate the assembly of actin filaments (reviewed by^3^). For example, it has been demonstrated that MT polymerization or de-polymerization induces activation of RhoGTPases signaling. Thus, MT-dependent Tiam1-expression led to Rac activation in neuron growth cones^42^. On the other hand, Trio and GEF-H1 (GEFs) bound to MT activated the GTPases pathway that led to neurite elongation^43–45^ or that could stimulate the formation of actomyosin fibres and facilitate the formation of focal adhesions^46^.

On the one hand, there is published evidence that demonstrates that RhoGTPases associate with lipid membranes and in doing so direct a number of molecular events that are essential for cell migration^47^. For example, it has been shown that fatty acids are important in retaining members of the RhoGTPase family in the plasmatic membrane, in a way that interrupting the de novo synthesis of fatty acids alters the levels of several lipids including cholesterol in the plasma membrane. This alteration in turn disrupted both, the activity and the recruitment of RhoA and Rac, generating an unexpected link between the metabolism of fatty acids and cell migration^48^.

A recent work by Marcial-Medina et al. demonstrated that OA acting on its two main receptors (FFAR1 and FFAR4) promoted migration of MDA-MB-231 and MCF-7 breast cancer cell lines via a mechanism that involves the recruitment of AKT and PI3K, with the participation of the epidermal growth factor receptor and the nuclear activation of NFkB^49^. The PI3K/AKT signaling pathway has been shown to control the activity of RhoGTPases in a variety of in vitro and in vivo models, including in processes of cell migration^50^.

Regarding the role of Cdc42, there is evidence that some extracellular signals recognized by the cells activate the RhoGTPase Cdc42 at the leading edge that in turn establishes a Par6–Par3–PKC polarity complex^51^. However, we did not observe that OA incremented the activity of Cdc42 differently at the front or rear of the cells. Therefore, more experiments should be carried out to either demonstrate or rule out a definite role of the polarity complex activated by Cdc42 in the OA-induced MTOC orientation.

Our findings provide a crucial insight on the effect of unsaturated FA on cell migration. We expect that cells exposed to varying concentrations of FA will be more susceptible to the effects of OA and LA. In particular, ovarian cancer cells as ovarian cancer (OC) metastasis occurs mainly in the peritoneal cavity where the coelomic fluid accumulates as a consequence of the intraperitoneal tumor growth causing ascitis^52^. In fact, ascites usually are enriched in FA; analysis of human ascites showed that OA content increases in malignancy compared with ascitic fluid from patients with cirrhosis^53^. Thus, an environment enriched in OA or LA could lead to a more efficient OC cell migration and dissemination within the peritoneal cavity. Therefore, we propose that the content of OA and LA in the ascitic fluid is a key factor that could be taken into consideration in the clinic. However, how specific FA or a combination of them influences the development of OC has been poorly investigated.

Abundant adipose tissue is present in the omentum^54^ which could be investigated as a source of lipids to the neighboring cells, opening an eventual therapeutic target (e.g., focal reduction of FA)^55^. Although semi-localized chemotherapies restricted to the abdominal cavity are now an established clinical practice^56–58^, these should be expanded to include local delivery of specific lipids alone or in combination. Therefore, more studies on dietary FA metabolism should be designed to modify lipid composition or metabolism with the aim of limiting the metastatic behavior of OC cells.

Because tumor cell migration is one of the main mechanisms of metastatic pathogenesis and since ALA, LA and OA are components of the peritoneal fluid in coelomic metastasis of OC^28^, we propose that understanding the regulation of migration by ALA, LA and OA will contribute to management of particular malignancies such as metastatic cancers. To achieve this goal, the effects of these FA on cancer cell migration should be examined in other cancer-derived cell lines and even in 3D models. More generally, understanding the effect of the ubiquitous FA on cell migration could shed light on complex processes such as embryonic development, wound healing and tissue regeneration.

In conclusion, in this study we show that LA and OA affect 2D cell migration via a modification of the cytoskeleton that results in a movement that is faster and straighter-orientated. We found that LA and OA treated cells: (1) protrude more filopodia than controls and these filopodia are highly motile; (2) treated cells have a dynamic lamellipodia at the front of migration that depends on activity of Rho GTPases, principally Rac and Rho; (3) orientate MT early towards the cell front and (4) increase the dynamics of MT. Put together, these changes result in cells that migrate in a more orientated and straightforward manner. Thus, this work provides evidence and demonstrates for the first time that unsaturated LA and OA increase cell migration rates in a model of ovarian cancer cells.

## Methods

### Cell culture

Human ovary cancer SKOV-3 cells were cultured in DMEM/F12 medium (Invitrogen) supplemented with 10% FBS (Natocor), 1% penicillin + streptomycin (10,000 units/ml + 10 mg/ml, Gibco) and HEPES 10 mM, at 37 °C, 5% CO_2_, in a humidified incubator. The SKOV-3 cells were generously donated by Dr. Osvaldo Podhajcer (Leloir Institute, Buenos Aires, Argentina). They were a passage 3 from an original, certified cell line acquired from ATCC (ATCC HTB77). The cell line was checked for Mycoplasma sp. by PCR according to Uphoff and Drexler^59^.

### Unsaturated fatty acids

Fatty acids α-linolenic acid (ALA, 18:3 ω3), linoleic acid (LA, 18:2 ω6) and oleic acid (OA; 18:1 ω9) (Nu-Chek Prep, Inc., Elysian, MN) were prepared under a nitrogen atmosphere and then stored at − 80 °C. Working solutions of 10 mg/ml (~ 35 mM) were diluted in ethanol and were handled on ice and in the dark.

### Wound healing assay

A scratch assay was carried out to study cell migration^17^. Briefly, SKOV-3 cells were grown to confluence over 24 h in tissue culture plates or glass depending on the experiment in 10% FBS DMEM/F-12. A linear scratch was made using a 200 μl tip. Each well was then washed with PBS 2 times and then filled with serum free DMEM/F-12, containing 16 μM of ALA, LA or OA. Ethanol was used as control as it is the diluent for the FA. We have established that ethanol at the concentration and exposure times used in our experimental setting did not alter cell survival, migration rate or cytoskeleton structures (data not shown). We used serum-free media in all experiments with FA for 4 main reasons. First, foetal serum contains undefined amounts of many different fatty acids^60–62^ a factor that in itself could contaminate our essays of the activity of individual FA. Second, serum contains non-negligible amounts of extracellular matrix proteins such as fibronectin and laminin that are well known to speed up and facilitate cell migration. In fact, serum is often used as a chemo attract and just for this reason^63^. Third, there is evidence that serum itself is capable of promoting cell migration in various systems and stimulate cell proliferation^64^. Finally, serum contains significant amounts of albumin, a carrier protein known to bind and interfere with the activity of FA^60,65,66^.

Cells were then incubated for 24 h at 37 °C, 5% CO_2_ in a humidified incubator until the experiment.

### Immunofluorescence

For actin and microtubule staining and mitosis quantification, cells were fixed in 4% PFA/sucrose for 20 min at room temperature, permeabilized with 0.2% triton- × 100 in PBS, and then blocked with 5% bovine serum albumin in PBS for 30 min. Immunostaining was performed by incubation with primary antibody diluted in 3% bovine serum albumin in PBS overnight at 4 °C, and followed by incubation with secondary antibodies for 1 h at room temperature. Phospho-Histone H3 (Ser10) (6G3) mouse monoclonal antibody (Cell Signaling Technology Cat# 9706, RRID:AB_331748), was used at 1/1000. Monoclonal anti alpha Tubulin antibody produced in mouse, clone DM1A (Sigma) was used at 1/500. Secondary DyLight 594 anti-mouse IgG (H + L), made in horse antibody (Vector Laboratories Cat# DI-2594, RRID:AB_2336412), was used at 1/500. Donkey anti-mouse IgG (H + L) highly cross-adsorbed secondary antibody, Alexa Fluor 488 (Invitrogen Cat# A21-202, RRID:AB_141607) was used at 1/500. TRITC-conjugated phalloidin (Sigma) was used at 0.1 mg/ml, and DAPI (Invitrogen) was used at 1/10,000. After washing 3 times 5 min each with PBS, immuno-stained cells were mounted with FluorSave (Calbiochem).

For quantitation of MTOC orientation cells were fixed with methanol at – 20 °C for 3 min after 0, 10, 30, 60, 90, 120, 180, 240 and 360 min after scratch and stained for alpha-Tubulin using the monoclonal anti alpha-Tubulin antibody produced in mouse, clone DM1A (Sigma) at 1/1000; anti gamma-Tubulin antibody produced in rabbit (Sigma Cat# T3320) (at 1/2000) diluted in 3% bovine serum albumin in PBS and incubated overnight at 4 °C. DAPI was used at 1/10,000 to label the nucleus. Secondary donkey anti-rabbit IgG (H + L) highly cross-adsorbed secondary antibody, Alexa Fluor 568 (Invitrogen Cat# A10042, RRID:AB_2534017), was used at 1/500 diluted in 3% bovine serum albumin in PBS.

### Cell viability assay

Cell viability was assayed using Resazurin (SIGMA-Aldrich) as previously described. 5 × 10^3^ SKOV-3 cells per well were plated, and either ALA, LA or OA FA were added next day in each well at 0, 4, 8, 16, 32, 64, 125, 250 μM in DMEM/F12 media without serum. 24 h later Resazurin (1 μg/ml) was added and cell incubated for 2 h at 37 °C, 5% CO_2_. Plates were read at 570–630 nm using a microplate reader (Glomax Biorad). This experiment was repeated with 4 separate cultures.

### Migration assay

For the scratch assay 7 × 10^4^ SKOV-3 cells were grown to confluence over 24 h in 6-well tissue culture plates in 10% FBS DMEM/F-12 and treated with FA as described above for ALA, LA, OA or control vehicle. Pictures of the scratch were taken 2 h (initial time, Ti) and 24 h (final time, Tf) after scratching. Images were taken under a phase-contrast microscope (Olympus IX81 epifluorescence inverted microscope). For quantification we used the images that showed a scratch of a width of 90 μm ± 5 μm at Ti. The migration for each experimental condition was calculated as the percentage of covered surface on the scratch at 24 h relative to the beginning of the assay (Ti, n = 10). Each experiment was repeated at least 4 times.

### Quantification of cell area and shape measurements during adhesion

SKOV-3 cells were treated with 16 μM of ALA, LA, OA or control vehicle for 24 h. Then cells were tripsynized and seeded on 96 well plates. After 10, 20, 40, 60 and 90 min cells were fixed in 4% PFA/sucrose and stained with phalloidin-TRITC and DAPI. Adherent cell counts were quantified using DAPI staining ateach time point. Cell area, perimeter and shape descriptors (circularity, roundness) were quantified by digital threshold analysis of actin stained structures using Fiji software (https://imagej.net/Fiji RRID:SCR_002285). Loss of circularity was reported as an indicator of progressive adhesion. Each experiment was replicated thrice.

Additionally to study cell adhesion, 5 × 10^3^ FA treated cells were seeded as explained above and washed after 10, 20, 30 and 40 min, then fixed in 4% PFA/sucrose. Adherent cells in individual wells were quantified by low magnification light microscopy. Each experiment was replicated 3 times.

### Stable cell lines

A SKOV-3 stable cell line was created by transfection with LifeAct-GFP plasmids using FuGene HD Transfection Reagent (Roche) according Wang et al.^67^. The SKOV-3 stable cell lineexpressingEB3-Cherry plasmid was generated using the polyethyleneimine (PEI) technique with 150 mM NaCl as diluent. Stably transfected cells were selected with 500 μg/ml of G418 (Life Technologies, Inc.) for 3 weeks. A G418-resistant clone was isolated, expanded and maintained on plates in complete media supplemented with 200 μg/ml of G418.

### Live-cell phase video microscopy

For live-cell video experiments, 5 × 10^3^ SKOV-3 cells were plated onto glass-bottom dishes (MatTek) and kept at 37 °C in a humidified incubator. The cell monolayer was treated with Either ALA, LA or OA (or control vehicle ethanol) as it was explained above. After the linear scratch was made on the cell monolayer cell were cultured for 16 h. Then cells were imaged every 15 min for a total of 6 h with controlled temperature at 37 °C during acquisition. Imaging was conducted employing a Zeiss Axiovert 200 microscope with a 10 × objective (ACHROSTIGMAT 10x/0.25 Zeiss) featuring a Hamamatsu Orca ER camera controlled by the Micro-Manager software. Individual cell trajectories were manually tracked using Fiji software (https://imagej.net/Fiji RRID:SCR_002285). using the center of the cell nucleus as the reference mark for cell translocation, as the nucleus translocates together with the cell body (Fig. 2a)^68^. Only cells that form the front line of migration were examined. Trajectory, displacement, straightness, orientation, cell polarity index, speed T (Trajectory), speed D (displacement), and polarized speed were calculated, integrating image analysis on Fiji and calculations on spread datasheets (https://imagej.net/Fiji RRID:SCR_002285). Cell trajectory was obtained by measuring the distance covered by the cell through measurements of the movement of the nucleus at every time point; displacement was defined as the vector from the initial position to the final position (using Draw Feret’s diameter Plugin); The co-linearity of the displacement angle respect to the optimal direction of wound closure (perpendicular to scratch) was defined as orientation and was calculated as *Cos*((displacement angle − wound closure angle)*π/180), ranging from -1 when migrating in opposite direction, to + 1 when perfectly aligned with the wound closure vector. Straightness was calculated as displacement/trajectory and ranged from 1 (linear trajectory) to near 0 (long trajectory). We defined a cell polarity index as expressed in this analysis as the product of straightness × orientation, and ranged from -1 to 1, with1 being a rectilinear migration in the best orientation, 0 a cell that does not advance to wound closure, and negative values for retracting cells. Migrating cell speed was calculated from trajectory (speed T) or displacement (speed D) over the time course of the experiment. Polarized speed was used as a final integrated indicator of cell migration, being the product of speed D × cell polarity index, as a measure of effective cell speed in terms of wound closing.

### Visualization of actin cytoskeleton structure and dynamics

In order to study actin cytoskeleton dynamics, the SKOV-3 LifeAct-GFP stable cell line was plated onto glass-bottom dishes (MatTek) and kept at 37 °C in a humidified incubator. Cells were incubated with LA, OA or control vehicle ethanol as described above. After the scratch, cells were cultured for 2–4 h and then were imaged every 3.7 s using an Olympus FV-1200 confocal microscope with a 60 × oil/silicone immersion objective (PLANAPO NA 1.42)on a thermostatized stage and 5% CO_2_ atmosphere. Images were acquired with the FluoView10-ASW software (RRID:SCR_014215), and then processed offline with Fiji software (https://imagej.net/Fiji RRID:SCR_002285).

### Fluorescence recovery after photobleaching (FRAP)

For FRAP assays, a scratch assay was conducted on a SKOV-3 LifeAct-GFP cells monolayer, in the presence of LA, OA, or control vehicle ethanol, as described above. An Olympus FV-1200 confocal microscope was employed. One pre-bleaching scan was acquired, followed by a photobleaching step using continuous full intensity 473 nm laser during 4 s, on a 10 μm^2^ circular region of interest (ROI) near the edge of the cell border. Sequential scanning at 400 ms intervals for 30 s followed recovery of fluorescence. FIJI Z-profiling was used to obtain the fluorescence levels over time in the different ROIs. Fluorescence values over time were subjected to offline background subtraction, were relativized to pre-bleaching levels, and were corrected by photobleaching during the scanning phase to allow the construction of FRAP curves. Data points rendered a single-exponential decay curve. Plateau values were used to estimate mobile-immobile fraction. A total of 8 experiments per condition were analyzed.

### Activity of Rho, Rac and CDC42 in migrating cells estimated by FRET probes

The FRET probes designed by Pertz and Matsuda were used to estimate the activity of the Rho, Rac1 and CDC42 GTPases in migrating cells incubated with OA or control^69–72^. These probes have two fluorophores, YFP and CFP, which in active conformation transfer the energy from YFP to CFP. Transient transfections were performed by the PEI method (using the plasmids bearing the different kinds of probe constructs), on confluent SKOV-3 cells. 24 h post transfection culture media was replaced by culture media without serum, with OA or control vehicle ethanol and then incubated overnight at 37 °C in a humidified incubator. The following day, a scratch wound was inflicted on the monolayer and 4–6 h later, cells were fixed in 4% PFA/sucrose. Next, monolayers were washed 3 times with PBS, and the glasses were mounted on MoWiol (Sigma). In order to make FRET activity maps, acquisition was performed on a confocal Olympus FV-300 Microscope, objective lens = PLAPON 60X oil/silicone immersion objective. Only cells on the first line of the scratch were imaged. Image acquisition was carried out in three ways: two for FRET calculations (YFP-CFP and CFP) and another one for improving segmentation of the cell image (YFP). FRET activity maps were elaborated in Fiji, using a semi-automatized macro that allowed personalized background correction and noise reduction/smoothing prior to further calculations (https://imagej.net/Fiji RRID:SCR_002285). The signal was the result of the FRET channel signal (YFP-CFP) divided by the CFP channel signal. The activity map was limited by border selection of the YFP image. A Thermal LUT was assigned to visually represent FRET activity, warm color was an indicator of high activity, white of medium activity, and the cool end of the spectra indicated low activity. Regions of interest in the leading edge and the rearof the cell were analyzed for each cell. Measurements were made on these regions to estimate and calculate the FRET absolute (FRONT or REAR) and relative activity (FRONT/REAR) in each cell for each probe. Each experiment included between 10 and 20 replicates with a total of 3 independent experiments. Cell image was excluded from the FRET calculation if it was out of focus, photobleached with high background, with another cell on top or isolated from the monolayer.

### MT orientation

For MT orientation analysis, a scratch assay was conducted on a SKOV-3 cells monolayer, treated with LA, OA, or control vehicle ethanol for 24 h, as described above. Cells were fixed with cool methanol after 0, 10, 30, 60, 90, 120, 180, 240 and 360 min after scratch and stained for alpha-Tubulin and/or gamma-Tubulin and DAPI. For polarity quantization the MTOC position was measured respect to the direction toward the front of migration. We considered a polarized cell if the MTOC and the MT were orientated towards the migration front with a deviation from a perfectly straight line of up to 60°. 5 fields per condition were selected and 20–40 cells per repetition were examined. Each experiment was repeated 4 times.

### MT dynamic

For MT dynamic analysis, a scratch assay was conducted on a SKOV-3 EB3-Cherry treated with LA, OA, or control vehicle ethanol for 24 h, as already described 2–4 h after scratch, cells were imaged every 400 ms for 90 s using an Olympus FV-1200 confocal microscope with a 60 × oil/silicone immersion objective (PLANAPO NA 1.42) on a thermostatized stage. Images were acquired with the FluoView10-ASW software (RRID:SCR_014215). Images were processed offline using a plusTipTracker from U-Trackin MatLab Version 1.1.4 (https://www.utsouthwestern.edu/labs/danuser/software), made by Danuser’lab^73^.

### Statistical analysis

Statistical tests, graphs, and curve fitting, were carried out in Prism 6.0. Charts or plots show mean (± SEM) values. Normal distribution of each data set was examined using D’Agostino-Pearson normality check. If data passed the test, Student t-tests were used for comparison between 2 treatments. For data that were not normally distributed, non-parametric tests were used (Mann–Whitney to comparison between 2 groups and Kruskal–Wallis with Dunn’s post-hoc test for multiple comparisons). Significance levels are as follows: *p < 0.05, **p < 0.01, ***p < 0.001 and ****p < 0.0001.

## Supplementary Information

Supplementary Video 1.

Supplementary Video 2.

Supplementary Video 3.

Supplementary Information 1.

Supplementary Information 2.
