# Supplementary figures and images for: Linoleic and oleic acids enhance cell migration by altering the dynamics of microtubules and the remodeling of the actin cytoskeleton at the leading edge
Source: Sci Rep. 2021 Jul 22;11:14984. doi: 10.1038/s41598-021-94399-8 (PMC8298526; doi:10.1038/s41598-021-94399-8)

**a**

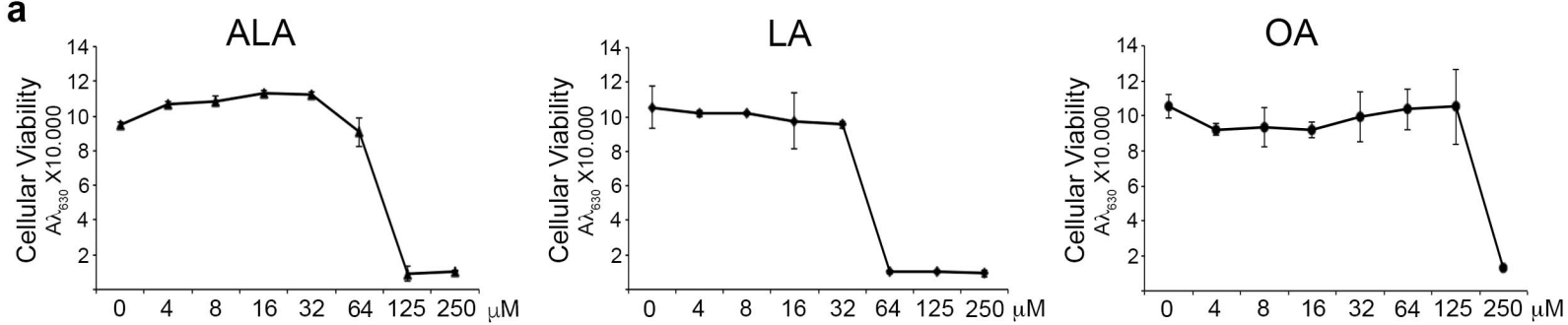

**b**

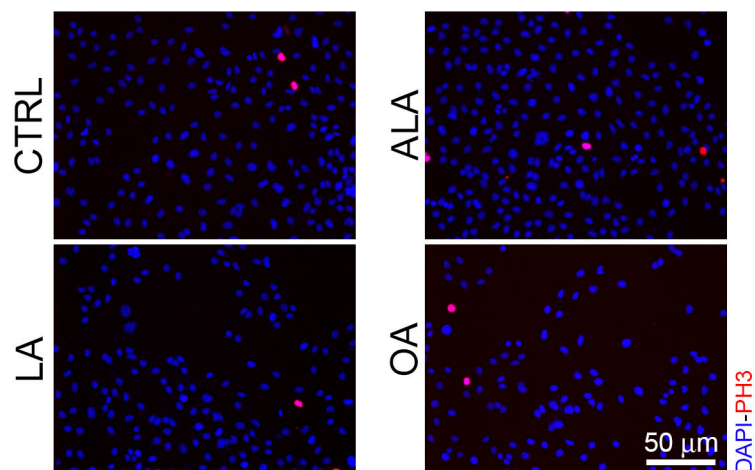

**c**

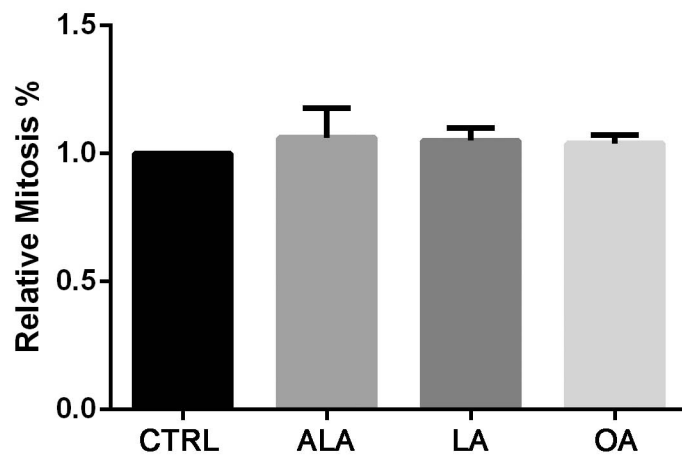

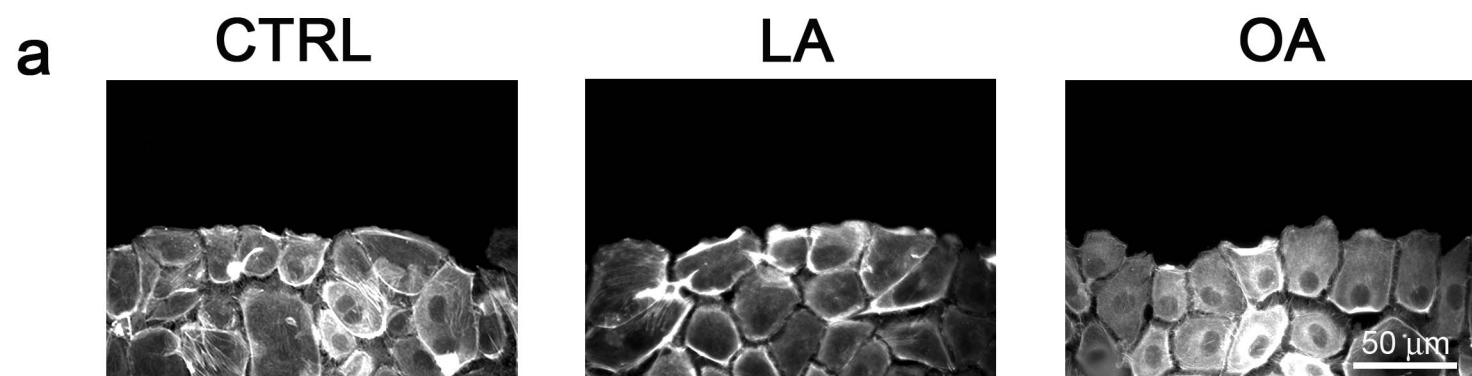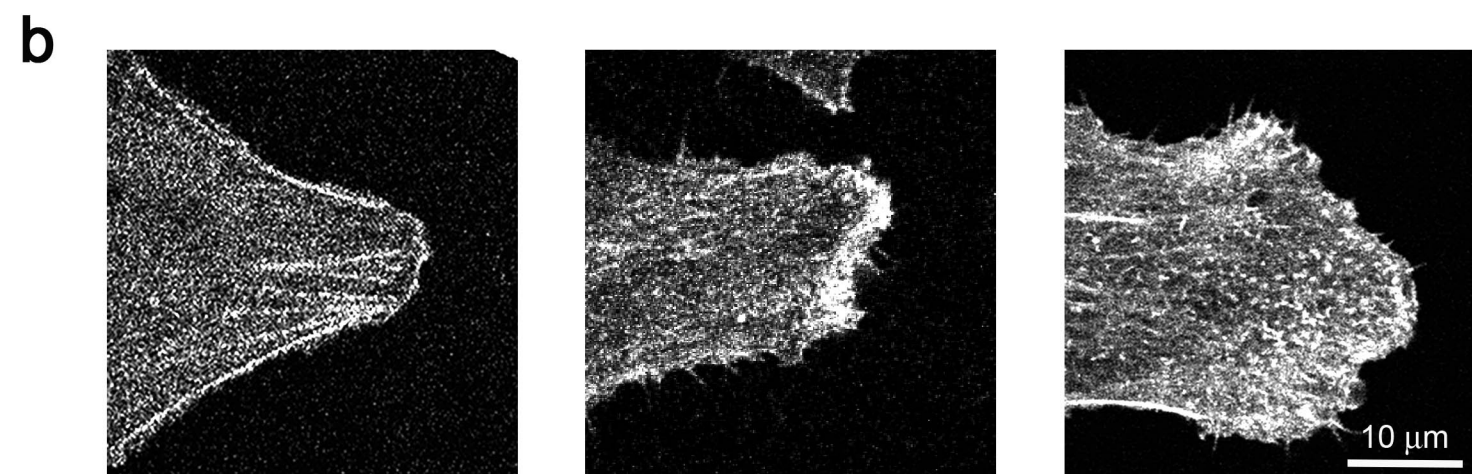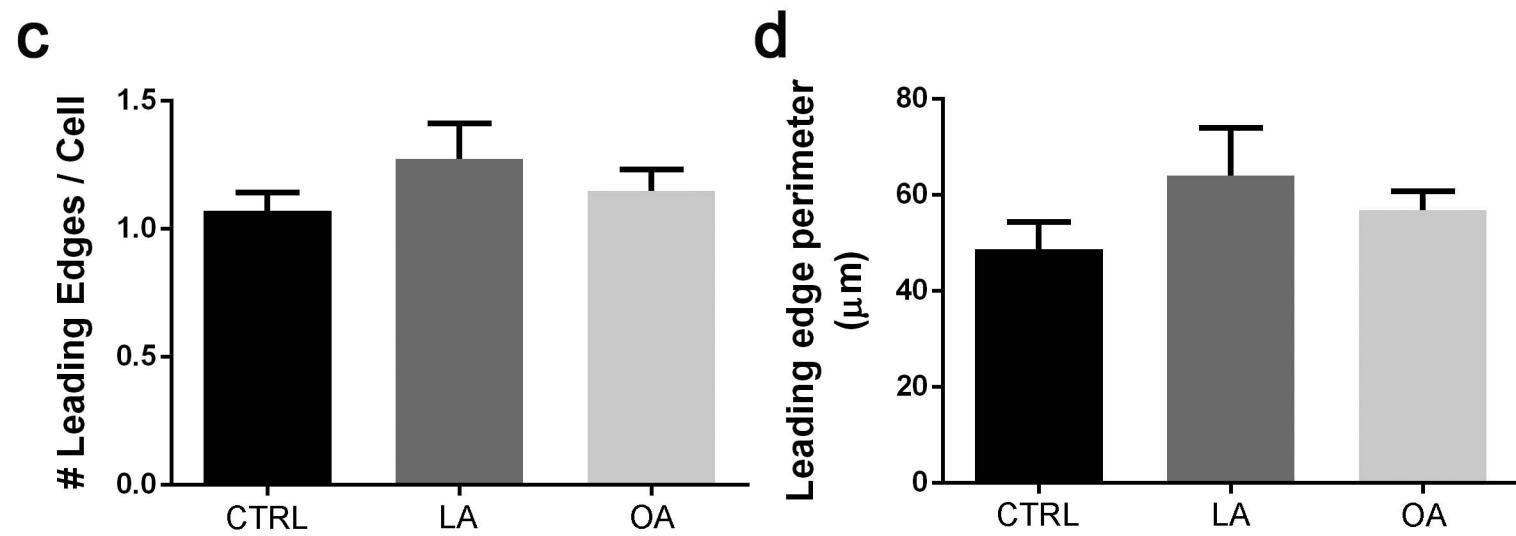

**a**

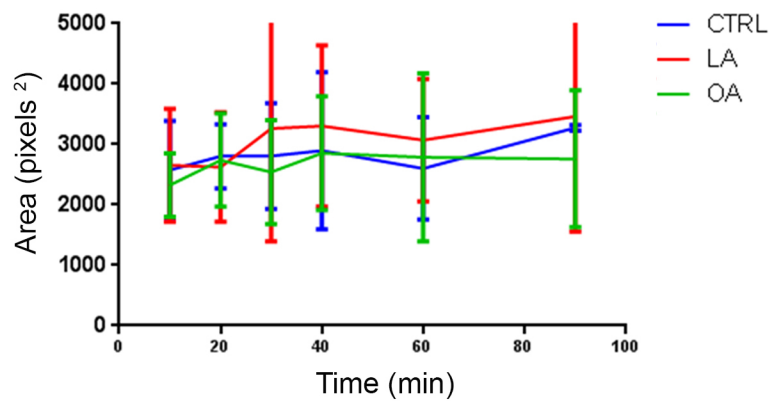

**b**

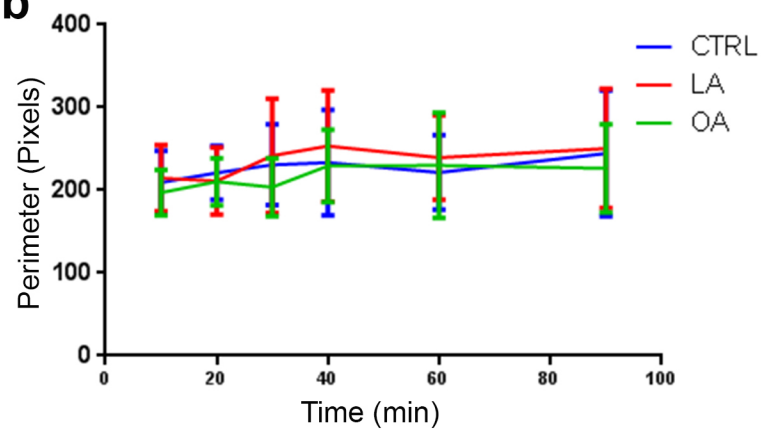

**c**

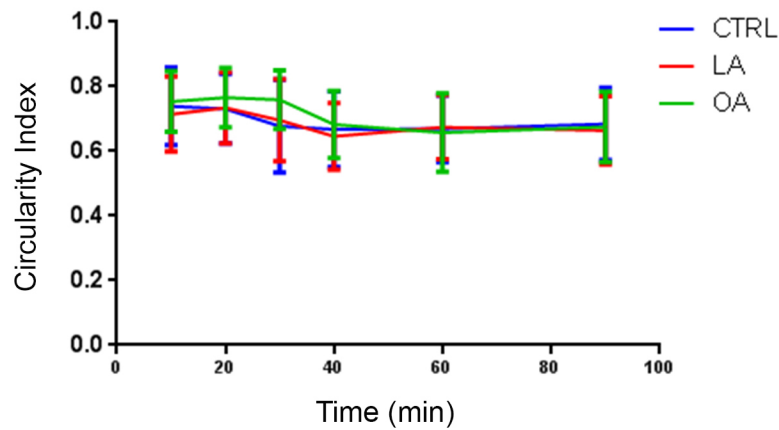

**d**

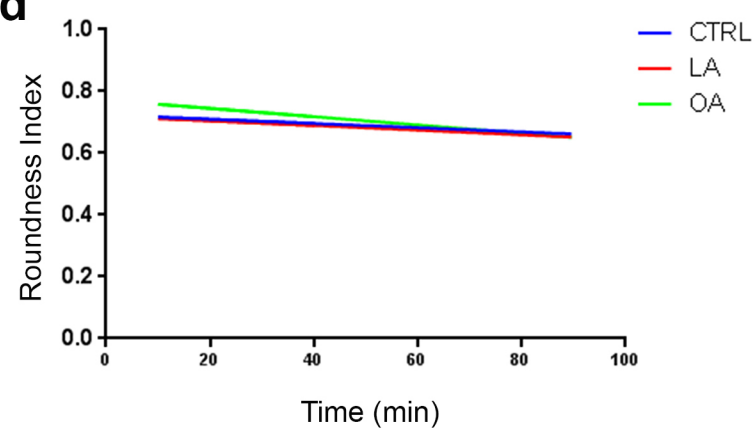

**e**

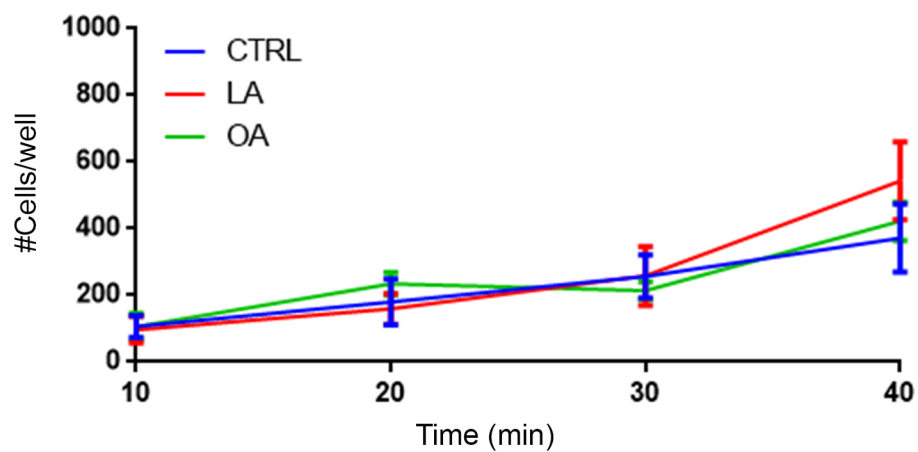

**a**

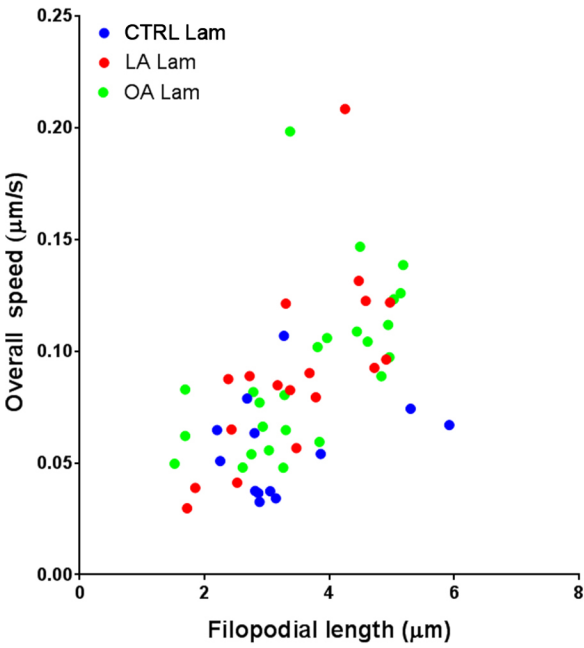

**b**

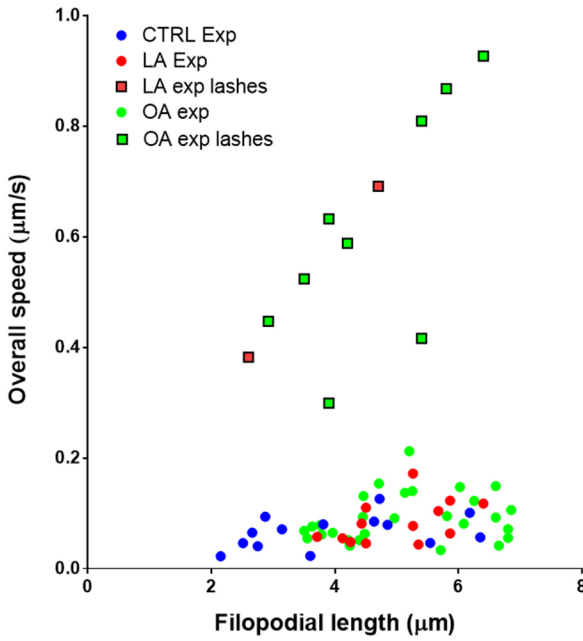

Supplement: Supplementary file 4 — Supplementary Information 1. [file 41598_2021_94399_MOESM4_ESM.pdf]
