# Supplementary material for: Linoleic and oleic acids enhance cell migration by altering the dynamics of microtubules and the remodeling of the actin cytoskeleton at the leading edge
Source: Sci Rep. 2021 Jul 22;11:14984. doi: 10.1038/s41598-021-94399-8 (PMC8298526; doi:10.1038/s41598-021-94399-8)
Supplement: Supplementary file 5 — Supplementary Information 2. [file 41598_2021_94399_MOESM5_ESM.docx]

SUPPLEMENTARY FIGURE LEGENDS

**Linoleic and Oleic acids enhance cell migration by altering the dynamics of microtubules and the remodeling of the actin cytoskeleton at the leading edge**

Masner M^1^, Lujea N^1^, Bisbal M^2^, Acosta C^3^ and Kunda P^1^*****

**Supplementary Figure S1. Addition of low dose (16 μM) unsaturated fatty acid to the culture media does not induce cell death or alterations in mitotic index.** (a) Viability curves of *in vitro* SKOV-3 cells in the presence of 0, 4, 8, 16, 32, 64, 125, 250 μM unsaturated FA ALA, LA or OA assayed by Rezarsurin. Data represent Absorbance at 570-630 nm. Viability curves were carried out in triplicates, the graph shows mean ± SEM.(b) Mitotic index among control, ALA, LA and OA treated cultures (16 μM) does not differ between treatments as evidenced by fluorescent staining with phosphoHistone-3 (Ser-10) mitotic marker. Mitotic index is represented as bars showing mean ± SEM, and statistical significance analyzed with Kruskal-Wallis test with Dunn's multiple comparisons, 10 random visual fields were inspected per condition in 2 independent experiments.

**Supplementary Figure S2.LA and OA did not affect morphometric patterns in SKOV-3 treated cells**

(a) Actin staining with phalloidin of SKOV-3 cells at 2h post scratch shows formation of lamellipodia at the cell front (facing the scratch) in control, LA and OA. Scale bar, 50μm. (b) SKOV-3 cells stably expressing LifeAct-GFP were used in confocal live cell videomicroscopy for analysis of cell morphology and actin structures. Photographs show filamentous actin at the leading edges of representative migrating cells of control, LA and OA at 4-6 h post treatment. Scale bar 10μm. The number of leading edges per cell (c) or the leading edge perimeter (d) did not differ significantly among treatments. Data analyzed using a Kruskal-Wallis test with Dunn´s post-hoc multiple comparisons, and shown as bars with mean ± SEM (n=15-20).

**Supplementary Figure S3.SKOV-3 cell spreading and adhesion does not differ after unsaturated fatty acid treatment.** SKOV-3 cells were exposed to serum free LA, OA or control for 24h. Later cells were tripsynized and seeded in 96 wells, then left to adhere. (a-d) Actin staining of tripsynised cells adhering to multi-wells was conducted at different times post seeding, and shape descriptors were measured by fluorescence imaging. Cell area (a) and perimeter (c) increases over time in all treatments as cells adhere. (b) circularity (dependent on both previous variables) decreases over time as cells adhere and make projections, but loss of circularity follows the same overall pattern of decay among all treatments. (d) Linear regression of circularity index showing a comparable decay of circularity among treatments. (e) Adherent cells were counted for each control, LA and OA by microscopic imaging. Cell counts at different times are shown as line graphs with no significant differences between them. Experiments were done in triplicates.

**Supplementary Figure S4.Speed and length correlation of lamellipodial and exploratory filopodia in LA and OA treated cells**. Dot plots of average speed vs. filopodial length are shown for lamellipodial (a) and exploratory (b) filopodia in control (blue), LA (red) and OA (green).

**Movie 1.**Time-lapse confocal videomicroscopy of SKOV-3 cells stably expressing LifeAct-GFP. A linear scratch on a cell monolayer was made using a 200 μl tip. After culturing for 4-6h cells were imaged every 3.7 s for 5 min using a Olympus FV-1200 confocal microscope with a 60x oil/silicone immersion objective. Images were acquired with the FluoView10-ASW software (RRID:SCR_014215), and then processed offline with Fiji software (https://imagej.net/Fiji RRID:SCR_002285).

**Movie 2.**Time-lapse confocal videomicroscopy of SKOV-3 stably expressing LifeAct-GFP cell treated with 16 μM LA. Cell monolayer was incubated with FAs as described in Methods then a linear scratch was made using a 200 μl tip. After culturing for 4-6h cells from the cells were imaged every 3.7 s for 5 min using an Olympus FV-1200 confocal microscope with a 60x oil/silicone immersion objective on a thermostatized stage and CO_2_ atmosphere. Images were acquired with the FluoView10-ASW software (RRID:SCR_014215), and then processed offline with Fiji software (https://imagej.net/Fiji RRID:SCR_002285).

**Movie 3.**Time-lapse confocal videomicrocopy of SKOV-3 stably expressing LifeAct-GFP cell treated with 16 μM OA. Cell monolayer was incubated with FAs as described in Methods then a linear scratch was made using a 200 μl tip. After culturing for 4-6h cells from the cells were imaged every 3.7s for 5 min using a Olympus FV-1200 confocal microscope with a 60x oil/silicone immersion objective on a thermostatized stage and 5% CO_2_ atmosphere. Images were acquired with the FluoView10-ASW software (RRID:SCR_014215), and then processed offline with Fiji software (https://imagej.net/Fiji RRID:SCR_002285).
